# Adoption of E2PLUS tools and resources to promote the development of institutional capacity for patient-centered and community-engaged research at a cancer center

**DOI:** 10.1017/cts.2025.27

**Published:** 2025-02-12

**Authors:** Katherine J. Briant, Prajakta Adsul, Elizabeth A. Carosso, Marty Chakoian, Diane Mapes, Terri Coutee, Bridgette Hempstead, Laurie Hassell, Wendy Law, Jason A. Mendoza

**Affiliations:** 1 Fred Hutch/University of Washington/Seattle Children’s Cancer Consortium, Seattle, WA, USA; 2 Department of Internal Medicine, School of Medicine, University of New Mexico and Cancer Control and Population Sciences Research Program, University of New Mexico Comprehensive Cancer Center, Albuquerque, NM, USA; 3 ZERO Prostate Cancer Support Group, Seattle, WA, USA; 4 Independent Patient Advocate, Seattle, WA, USA; 5 DiepCfoundation, Duvall, WA, USA; 6 Cierra Sisters, Inc, Renton, WA, USA; 7 Institute of Translational Health Sciences, University of Washington, Seattle, WA, USA

**Keywords:** Patient and community-engaged research (P/CEnR), community-academic partnerships, health equity, patient advocates, qualitative methods, academic health center

## Abstract

**Introduction:**

The Fred Hutch/University of Washington/Seattle Children’s Cancer Consortium’s (Consortium) Office of Community Outreach & Engagement (OCOE) joined Stanford Medicine and Morehouse School of Medicine in implementing Engage for Equity Plus (E2PLUS), a multi-institutional community of practice to learn and share patient-centered and community-engaged research (P/CEnR) practices. University of New Mexico (UNM) facilitated this collaboration.

**Methods:**

The Consortium formed a Champion Team of 12 people who participated in two virtual workshops facilitated by UNM. Consortium executive leadership (*n* = 4) participated in interviews, and investigators (*n* = 4) and community members/patient advocates (*n* = 8) participated in focus groups to provide institutional context regarding P/CEnR. This is a paper on the process and findings.

**Results:**

Through E2PLUS engagement, the Champion Team identified four strategies to address institutional health inequities: 1) increase participation of underrepresented groups at all levels of institutional leadership and advisory boards; 2) create an Office of Patient Engagement to train and support patients who participate in institutional initiatives and advise research teams; 3) expand community engagement training, resources, and institutional commitment to focus on community-identified social and health needs; and 4) establish an umbrella entity for health equity efforts across the Consortium.

**Conclusion:**

While the Consortium had longstanding community advisory boards and faculty and staff with P/CEnR expertise, it did not have centralized and institutionally supported P/CEnR resources, policies, and infrastructure. By participating in E2PLUS, the Champion Team received technical assistance to leverage qualitative data to influence strategies to guide the development of Consortium health equity infrastructure and capacity for P/CEnR in Washington.

## Introduction

The Fred Hutch/University of Washington/Seattle Children’s Cancer Consortium (Consortium), the only National Cancer Institute (NCI)-designated Comprehensive Cancer Center in Washington State (WA), consists of three partners: Fred Hutchinson Cancer Center (Fred Hutch), University of Washington (UW), and Seattle Children’s. These partner institutions collaborate to engage in the full spectrum of cancer research programs to reduce the burden of cancer among populations in WA. The Consortium’s Office of Community Outreach & Engagement (OCOE) uses a data-driven approach to identify WA’s cancer-related needs and develop strategic research aims to address them [1,2].

The OCOE applies community-based participatory research (CBPR) principles to reduce the cancer burden in WA through an equitable, bidirectional approach in outreach, education, and programing. CBPR is a strategy based on the principle that issues are much more likely to be solved if individuals and communities are involved in the process of addressing them [3,4]. This is especially true for communities experiencing health disparities [4,5]. Among the underserved/marginalized, CBPR increases the acceptability and relevance of the health topic, the recruitment and retention of study participants, the likelihood of producing a change in the population, and the study’s reach by enhancing dissemination of findings to relevant groups [5,6]. CBPR enables the equitable bi-directional exchange of knowledge between researchers and community members [4].

The University of New Mexico (UNM) created Engage for Equity (E2) as a set of tools and resources to help community–academic research partnerships enhance and advance power sharing in health equity work [7,8]. UNM enhanced the E2 toolkit into Engage for Equity Plus (E2PLUS) to leverage the use of Champion Teams comprised of community-engaged faculty, community partners, and patient advocates [9]. The Consortium’s OCOE joined the E2PLUS project [9], facilitated by UNM, to collaborate with colleagues at UNM, Stanford Medicine (Stanford) in California, and Morehouse School of Medicine (MSM) in Georgia to learn and share best practices regarding patient-centered and community-engaged research (P/CEnR). For NCI-designated cancer centers like the Consortium, there is an urgency to adopt P/CEnR. The National Institutes of Health (NIH) is mandated to ensure the inclusion of women and members of racial and ethnic minority groups in all NIH-funded clinical research in a manner that is appropriate to the scientific question under study [10].

Experiences from the COVID-19 pandemic to the nation’s reckoning with racial inequities amplified the urgency of evolving the Consortium’s mission towards cancer equity. Consortium leadership recognized that an active commitment to communities is required *first* before attempting to engage them in research. While P/CEnR approaches are integral to OCOE’s program and research priorities, they are not systematically used by research teams throughout the Consortium partner institutions.

The Consortium’s participation in E2PLUS overlapped with several key initiatives. In 2020, Fred Hutch created an Office of Diversity, Equity and Inclusion (DEI) and appointed a Vice-President and Chief DEI Officer. In fall of 2021, ongoing dialogue within the UW led to the launch of the Center for Anti-Racism and Community Health within the UW School of Public Health (SPH). In early 2022, the Consortium launched a 6-month Task Force to examine inclusion and equity in research to identify patient-centric barriers to participation in clinical trials. In spring 2022, the Consortium was undergoing a restructuring of institutional partners as follows: 1) Fred Hutchinson Cancer Research Center (FHCRC) and Seattle Cancer Care Alliance (SCCA) were integrated to form the new Fred Hutchinson Cancer Center (Fred Hutch), an independent nonprofit organization, and 2) adult cancer care for UW Medicine and Fred Hutch became integrated and managed by Fred Hutch across their clinical sites. Finally, in July 2023, House Bill 1745 was passed in WA to improve diversity in clinical trials [11]. (See Discussion section.)

Joining E2PLUS provided the Consortium’s OCOE an opportunity to strengthen internal and external relationships and for introspection on how to address institutional and structural racism. The Consortium had several elements of P/CEnR, including longstanding community advisory boards and faculty and staff with P/CEnR expertise. However, the Consortium did not have centralized and institutionally supported P/CEnR resources and infrastructure, nor policies to encourage bidirectional community engagement in research and programs.

The timing of the key initiatives mentioned above, and participation in E2PLUS provided opportunities to influence health equity-oriented research and care. The E2PLUS community of practice with UNM, Stanford, and MSM offered peer support and guidance. The Consortium’s participation in E2PLUS interviews, focus groups, and workshops helped the Champion Team advance targets of change for action to strengthen equity-based P/CEnR. In this report, we present qualitative data from interviews, focus groups, and workshops from our engagement in E2PLUS.

## Materials and methods

UNM worked with three partners across the country (the Consortium, Stanford, and MSM) to scale up their evidence-based E2 toolkit. UNM facilitated E2PLUS to help partners develop practices to address institutional barriers using evidence-based metrics, workshops, and tools. OCOE participated in E2PLUS and worked with a Champion Team of 12 people (Table 1) to assess and develop research capacity for community-engaged research, with emphasis on underrepresented (UR) populations. The Champion Team included community members and patient advocates at the local and national level, as well as faculty, leadership, and staff who hold positions with Fred Hutch, Seattle Children’s, the UW School of Medicine, the UW School of Nursing, and the UW SPH. (At the time, Fred Hutch was known as FHCRC.)

**Table 1.** Consortium champion team roles & affiliations

Role	Affiliations
***Patient advocates/community members***	
Cancer survivor and patient advocate	ZERO prostate cancer
Cancer survivor and patient advocate	DiepCfoundation
Cancer survivor and patient advocate	Cierra Sisters, Inc.
Cancer survivor and patient advocate	Independent patient advocate
***Community engagement faculty***	
Director & associate professor	University of Washington School of Public Health Center for Anti-Racism and Community Health
Associate professor, public health sciences	Fred Hutch Cancer Center
Associate director of community outreach and engagement	Fred Hutch/University of Washington/Seattle Children’s Cancer Consortium
***Cancer center leader***	
Associate director of administration	Fred Hutch/University of Washington/Seattle Children’s Cancer Consortium
***Clinical and translational science award program***	
Director of community engagement	Institute of Translational Health Sciences, University of Washington
***Office of community outreach & engagement staff***	
Director of operations and partnerships	Fred Hutch/University of Washington/Seattle Children’s Cancer Consortium
Program administrator for research operations	Fred Hutch/University of Washington/Seattle Children’s Cancer Consortium
Project manager for indigenous cancer health equity initiative	Fred Hutch/University of Washington/Seattle Children’s Cancer Consortium

**Figure 1.**
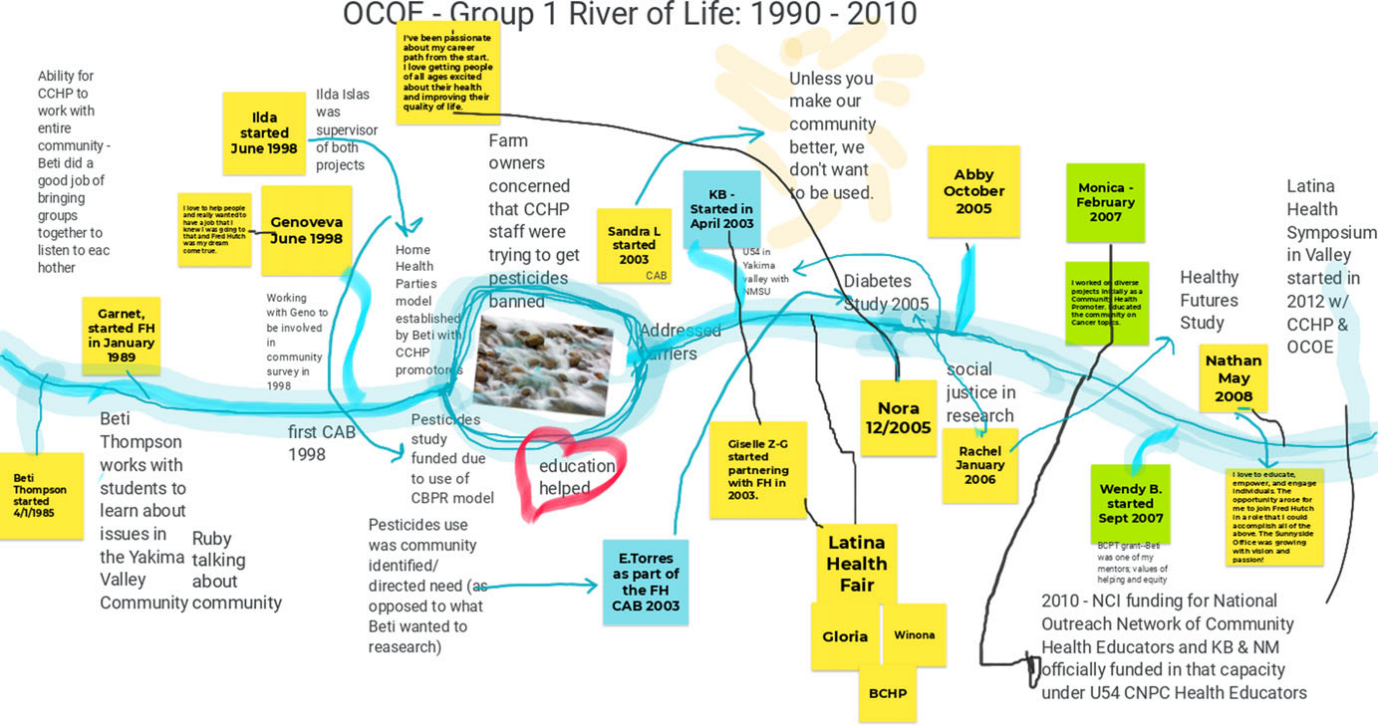
Office of Community Outreach & Engagement (OCOE) river of life (Group 1: 1990–2010). Abbreviations in Fig. 1: Center for Community Health Promotion (CCHP); K. Briant (KB); Fred Hutch (FH); community advisory board (CAB); Breast & Cervical Health Program (BCHP); National Cancer Institute (NCI); Community Network Program Center (CNPC).

**Figure 2.**
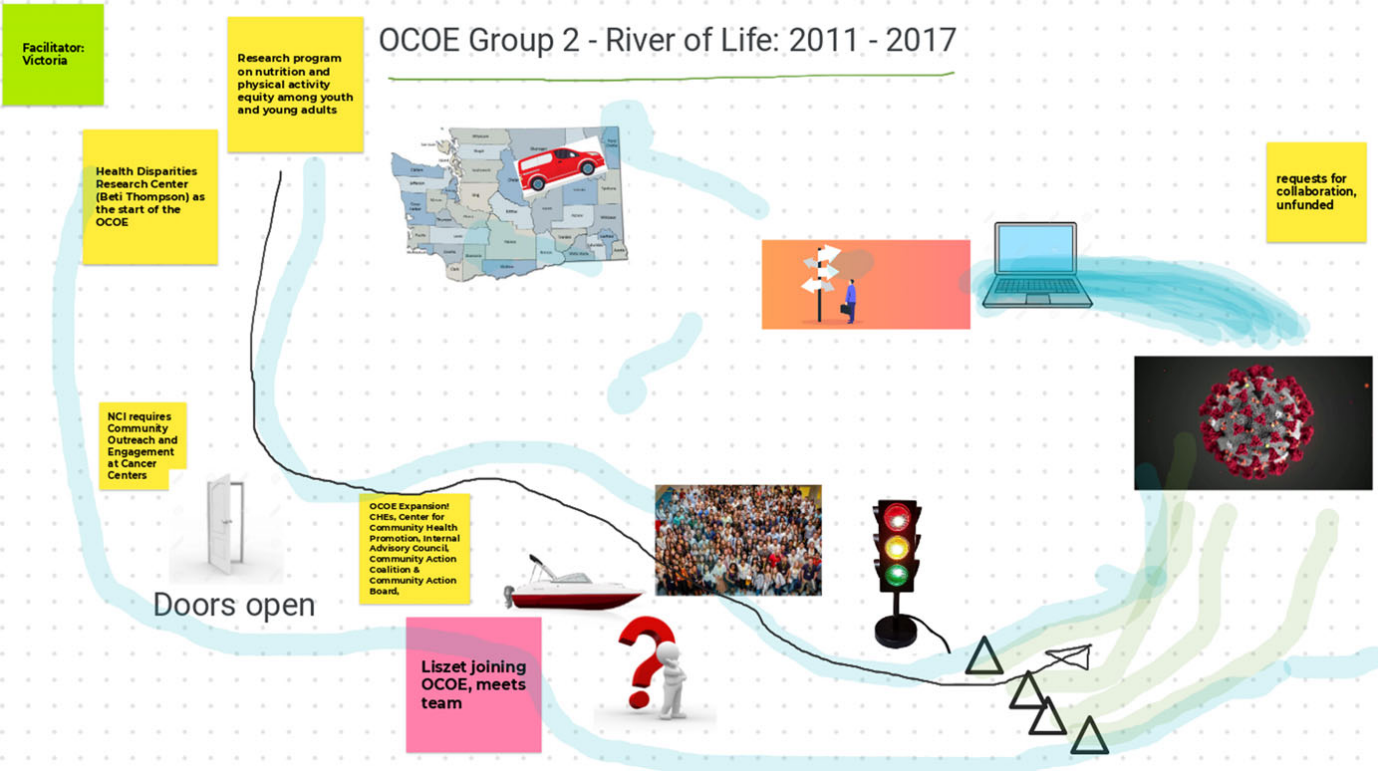
Office of Community Outreach & Engagement (OCOE) river of life (Group 2: 2011–2017). Abbreviations in Fig. 2: National Cancer Institute (NCI); community health educators (CHEs).

**Figure 3.**
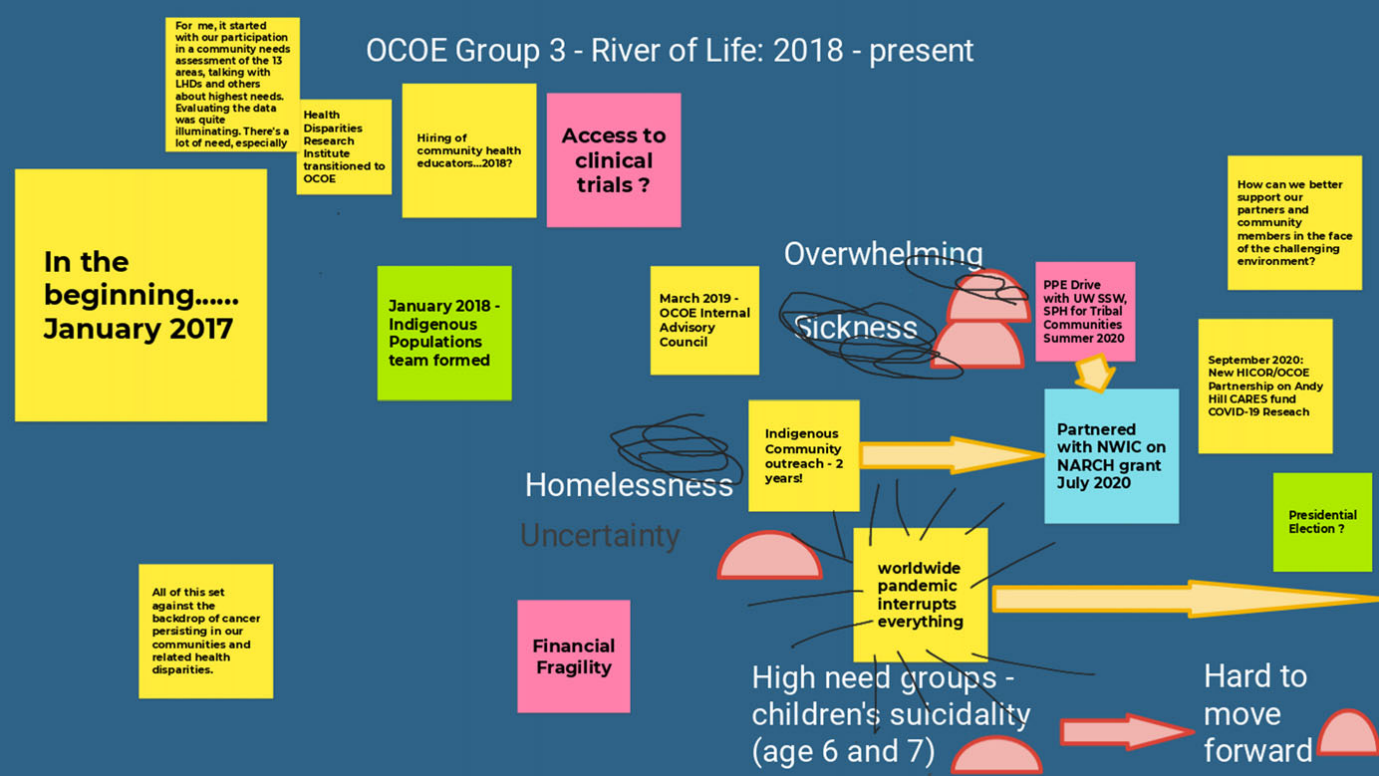
Office of Community Outreach & Engagement (OCOE) river of life (Group 3: 2018–present). Abbreviations in Fig. 3: personal protective equipment (PPE); University of Washington (UW); School of Social Work (SSW); School of Public Health (SPH); Northwest Indian College (NWIC); Native American Research Centers for Health (NARCH); Hutchinson Institute for Cancer Outcomes Research (HICOR); Andy Hill Cancer Research Endowment Fund (Andy Hill CARE fund); coronavirus disease 2019 (COVID-19).

Through E2PLUS virtual workshops facilitated by UNM, the Champion Team collaborated with other Consortium stakeholders to identify opportunities for change. The first virtual workshop, held over 2 days in the fall (November 1, 2021 (*n* = 23) and November 2, 2021 (*n* = 20)), used the E2 tool of the “River of Life” [12]. This reflective exercise helped participants validate and recognize the history and context of facilitators and barriers for P/CEnR at the Consortium by documenting a historical timeline with text, images and drawings while acknowledging barriers and milestones along the journey. Participants split up into three groups to put together the “River of Life” for three periods of time. Group 1 worked on the 1990–2010 time period; Group 2 worked on the 2011–2017 time period; and Group 3 worked on the 2018 to present time period (Figures 1–3).

In December 2022, UNM conducted virtual interviews with executive leadership (*n* = 4) of each Consortium institution to hear about these leaders’ vision and their assessment of challenges and possibilities for institutional change around health equity. The interview included questions such as, “These days there is a lot of emphasis on institutional transformation or innovation in health science. How do you envision institutional transformation or innovation for your institution?” and “What changes would you like to see happen in the next year to strengthen equity-based patient and community engagement as a sustained committed effort?” Leaders interviewed included the President and Director of FHCRC, President of SCCA, Chief Executive Officer of UW Medicine/Dean of UW School of Medicine, and Chief Academic Officer of Seattle Children’s Hospital. Interviews ranged from 30 to 45 minutes.

In addition, UNM conducted one virtual investigator focus group (*n* = 4) and one virtual community member/patient advocate focus group (*n* = 8) to assess similarities and differences among the stakeholder groups. The investigator focus group included questions such as, “How do you perceive the institution values P/CEnR?,” “In your opinion, what are the key barriers to operationalizing equity-based P/CEnR in your institution?,” and “What changes would you like to see happen in the next year to strengthen equity-based P/CEnR as a sustained committed effort?” The community member/patient advocate focus group included questions such as, “From your perspective, how do you think the institution values P/CEnR? How is this shown?” Focus group participants were recruited by Champion Team members. Focus groups were about 90 minutes in length.

Interviews and focus groups were recorded and transcribed. Data were analyzed collaboratively using Dedoose software [13] by a team of four people (including PA) with training in qualitative analysis. Using a consensus-based approach in group meetings, the analysts agreed on a set of themes and used these to create an institutional memo report. For each theme, there were associated quotes from participants (See Results).

The second virtual E2PLUS workshop was conducted over 2 days in the winter of 2022 (January 31, 2022 (*n* = 27) and February 28, 2022 (*n* = 25)). The purpose of these two workshop days was two-fold. During the first day, the qualitative interview and focus group data were presented back to participants. During the second day, participants engaged with the E2 tool of “CBPR Model Visioning” [12] process to reimagine and select specific targets of change for action to strengthen equity-based P/CEnR over the next 6 months.

UNM met with the Champion Team regularly to support identified targets of change and to provide summary reports to help the Champion Team further their agenda for change.

## Results

### E2PLUS workshops

During the first E2PLUS workshop held in the fall, group discussion focused on the history of CBPR and P/CEnR. Within the Consortium, P/CEnR began in the 1990s with a professor with dual appointments at Fred Hutch and UW, Dr. Beti Thompson, whose work established infrastructure to conduct CBPR in the central and western parts of WA [14–20]. Participants discussed facilitating factors to conducting P/CEnR, such as funding that calls for community engagement; having a workforce representative of the communities being served; and working with investigators who understand the importance and value of community as partners in research. Due to the length of time it takes to build trust and partnerships, participants also recognized challenges to P/CEnR, such as funding mechanisms that wax and wane or grant deadlines that don’t align with time needed to coordinate applications with community. There was also a lack of overarching structure and coordination for authentic community engagement across Consortium programs.

The E2PLUS winter workshop was scheduled over 2 days 1 month apart. The first day focused on discussion around the qualitative data results for the Consortium. (See Qualitative Data below.) There was discussion about how different entities (community members vs. researchers vs. institutional leaders) approach P/CEnR from different starting points and toward different outcomes and return on investment (short term of increasing diversity in clinical trials vs. long-term investment of engaging and building relationships with diverse communities to collaboratively address diversity in clinical trials).

The second winter workshop day focused on developing specific next steps and identifying outcomes. A major focus of the discussion centered on the lack of UR community participation on Consortium committees and processes as a structural barrier to cancer health equity. With that in mind, the Champion Team identified four strategies to address urgent and persistent institutional health inequities: 1) increase participation of UR groups at all levels of institutional leadership and institutional advisory boards; 2) create of an Office of Patient Engagement, with a major focus on UR patients, to train and support patients who participate in institutional initiatives and advise research teams as patient advocates; 3) expand community engagement training, resources, and institutional commitment, including nonresearch engagement focused on community-identified social and health needs; and 4) establish an umbrella entity for health equity efforts across the Consortium.

### Qualitative data

During the second E2PLUS workshop, UNM presented the qualitative data (leader interviews and investigator and community member/patient advocate focus groups) from the Consortium.

#### Executive leadership interviews

The individual interviews with executive leadership generated some common themes. First was recognition of the current state of practice around P/CEnR. Current practices identified 1) community engagement and health equity are not yet fully adopted as an overarching long-term vision; they may happen for specific projects, grants, or funding opportunities but are not part of the institutional research culture; 2) focus is on short-term recruitment of diverse populations in clinical trials; and 3) there are developing commitments to change institutional structures.

Second, interviews identified opportunities for change. Those identified included 1) listening to communities and having their insights inform Consortium work with a long-term commitment to community health and 2) institutionalizing how to recruit community members to make it a clear, sustainable, and equitable process that results in better representation in the workforce, advisory roles, research, and clinical trials.

*“I think the new generation is demanding this of us. My students and postdocs and doctors are not going to sit tight if we’re not addressing community engagement and we’re not thinking about how to involve different communities in our research.”*

*“I think the will is there and we’ve done all the easy stuff of having committees and training [of faculty/staff] we’re at the point now where we’re doing the hard work and over the next year, we’ll see where it goes.”*

#### Investigator focus group

The investigator focus group included those with a range of community engagement experiences from beginner to advanced. Similar to an outcome of the Champion Team’s River of Life exercise, there was recognition for the CBPR groundwork done by Dr. Beti Thompson. Investigators also recognized the OCOE as a resource for assisting with P/CEnR.

*“I think we, for the first time, engaged OCOE for this pilot study this year. So, we’re new to leveraging these connections. It’s been great. {I’m a clinician} and so interactions I have in that realm I think are different in many ways from feedback and info we’ve been able to get in our meetings this year to the OCOE. And so, I think we’re on the early side of things but I’m starting to sense the kind of information and how our projects will be somewhat different and the biases are already impacting my interactions and OCOE enabled me to take a step back.”*

Investigators shared that while they need to focus on project and grant specific requirements, such as IRB and translation of study documents, it is important to have institutional pathways to strengthen P/CEnR. They identified two strategies to better engage with patients and community members to conduct P/CEnR: 1) access to translators and interpreters for research, not just clinical care, and 2) being proactive by taking time to listen to patient and community voices to inform their work instead of reacting when issues arise and are voiced by patients and community members.

*“It was a little easier to get translators for clinical work, but not as easy for research, so that’d be really helpful.”*

*“I’d agree that much of the change is patient driven in a manner that is great because they’re the ones experiencing it, so having their say and their voice in it is important but that is a really reactive approach to change because if they’re already voicing opinions on it - it’s typically negative vs. having that proactive approach.”*

#### Community member/patient advocate focus group

The Community Member/Patient Advocate Focus Group offered a range of valuable perspectives. Members mentioned that there has been forward movement in P/CEnR, but they had concerns about disparities in cancer care and the community voice not being sought out or heard within the research enterprise. They also brought up power dynamics and how that impacts their voices being heard as well as their ability to get involved.

*“About researchers and doctors, you cannot understand what they’re talking about so therefore you cannot challenge what they’re talking about, because they hide behind the fact that they got these policies in place. Because they’re talking from their pedestal, they are not actually talking to change anything, they’re talking to bolster what they believe is how the outcome of their research is going to take place.”*

Members expressed that if a research project does not involve patient and community voices, it will be flawed. Since cancer impacts everyone, they added that bringing in family members and caretaker voices is also beneficial.

*“The institution of Fred Hutch or the Cancer Consortium, has all kinds of lectures, talk after talk, but I have never seen a patient talk. The only time patients are included on any kind of talk or a panel is once a year, when they have a special cancer patient panel and then we’re just lined up to tell our sad story.”*

Members mentioned that training on both sides (patient/community and research/provider) to encourage collaboration would be beneficial. They added that patient advocates should be compensated for their expertise and time.

*“The one thing we can do to help deal with both disparities and try to get a greater diversity of cancers involved is training the researchers to make those people feel welcome, actively seeking out their opinions.”*

*“You definitely need patient advocates who are trained, and then you can go back to their communities and explain to the people in their communities, what these clinical trials are and why they are important. I think, on the other flip side of that, if you are going to have patient advocates, they definitely need to be compensated because this is hard work.”*

The members of the group expressed appreciation in hearing common issues from each other during the focus group and wanted to continue having an opportunity to meet for mutual support and learning.

### Fred Hutch Task Force on Equity and Inclusion in Research

The 6-month Task Force was charged with examining current practices and resources around ensuring diversity and inclusion in research. This work was carried out by six working groups. Seven Champion Team members were invited to be part of the Task Force and two additional Champion Team members who are patient advocates were invited to present to the Task Force. The Task Force provided recommendations to improve efforts in seven areas that align with qualitative data gathered through E2PLUS (Table 2).

**Table 2.** Alignment between Engage for Equity PLUS (E2PLUS) qualitative data, Champion Team strategies, and director’s Task Force on Inclusion and Equity in Research recommendations

E2PLUS data	Champion Team strategies	Director’s Task Torce recommendations
Training researchers to make people feel welcome (C/P) Institutionalize success from grants into practice, such as patient navigators (I) More patient navigation and a clinical trials liaison program (L)	Increase participation of underrepresented groups at all levels of institutional leadership and advisory boards	Improving patient access: Ensure appropriate support to meet needs of patients and to connect patients to support reliably and in a timely manner
Researchers who take patients seriously and value patient opinions (C/P) Pathway for investigators to connect with community members who are interested in research (I) Develop clear, sustainable, effective institutional processes for investigators to reach out to communities (L)	Create an Office of Patient Engagement to train and support patients who participate in institutional initiatives and advise research teams as patient advocates Expand community engagement training, resources and institutional commitment to focus on community-identified social and health needs	Understanding patient and study participant needs: Better understand the needs/beliefs/values/gaps of patients and study participants, especially underrepresented minorities and develop strategies to meet needs
Trained patient advocates who can go back to community to explain clinical trials in lay terms (C/P) Getting translation/interpretation services for research (I) Creating more settings for patients/community members to get involved research and inform research (L)	Create an Office of Patient Engagement to train and support patients who participate in institutional initiatives and advise research teams as patient advocates Expand community engagement training, resources and institutional commitment to focus on community-identified social and health needs	Robust clinical trials infrastructure to promote diversity: Strengthen institutional infrastructure to enable inclusion and equity
Any research project that doesn’t involve patient/community voices is flawed (C/P) Consideration for relevant study participant incentives for community members (I) Developing commitment to changes in institutional structures such as cluster hire of underrepresented scholars (L)	Increase participation of underrepresented groups at all levels of institutional leadership and advisory boards Create an Office of Patient Engagement to train and support patients who participate in institutional initiatives and advise research teams as patient advocates Expand community engagement training, resources, and institutional commitment to focus on community-identified social and health needs. Establish an umbrella entity for healthy equity efforts across the Consortium	Study design and implementation: Encourage studies to be designed and implemented with equity in mind
Training for both patients and researchers would be beneficial (C/P) Need for institutional templates and pathways to strengthen P/CEnR (I) Tracking equity metrics in dashboards to follow clinical cancer outcomes (L)	Create an Office of Patient Engagement to train and support patients who participate in institutional initiatives and advise research teams as patient advocates Expand community engagement training, resources and institutional commitment to focus on community-identified social and health needs	Metrics and monitoring: Ensure studies are able to recruit a range of diverse participants by monitoring enrollment in real time and connecting studies to resources to bolster recruitment when needed
Compensation for patient advocates (C/P) P/CEnR is important for healthcare. More communication between clinical, basic, and public health researchers is needed to demonstrate how P/CEnR contributes to better science. (I) Community engagement mandates exist within specific projects/grants but are not mandated institutionally (I)	Increase participation of underrepresented groups at all levels of institutional leadership and advisory boards Create an Office of Patient Engagement to train and support patients who participate in institutional initiatives and advise research teams as patient advocates Expand community engagement training, resources and institutional commitment to focus on community-identified social and health needs Establish an umbrella entity for healthy equity efforts across the Consortium	Operational needs: Identify areas in our system that could be more inclusive and equitable in operations and address gaps and needs in the short term
The Consortium hosts many experts as speakers, but they are rarely patients. Power dynamics cause a huge gap (C/P) Be proactive in listening to community voices and making changes to help, rather than being reactive (I) The driving force is around recruitment in clinical trials rather than commitment to communities over time. How do we get better about listening to community and doing work based on their insight? (L)	Increase participation of underrepresented groups at all levels of institutional leadership and advisory boards Create an Office of Patient Engagement to train and support patients who participate in institutional initiatives and advise research teams as patient advocates Expand community engagement training, resources and institutional commitment to focus on community-identified social and health needs Establish an umbrella entity for healthy equity efforts across the Consortium	Relationships with community: Ensure that cancer prevention and care as well Consortium research are accessible to everyone in the community, and the Consortium is seen as a trusted partner in the community

**KEY:** C/P = community/patient focus group; I = investigator focus group; L = leadership interviews.

## Discussion

Participation in E2PLUS provided the opportunity for the Consortium to develop short-, medium-, and long-term outcomes. In the short term, a Champion Team with Consortium faculty and staff, patients, and community members was formed to mobilize this work. In addition, UNM conducted an analysis of institutional barriers and facilitators to P/CEnR through interviews and focus groups [21]. In the medium term, the Champion Team received technical assistance from UNM to develop an action plan with four strategies, based on the data, to drive institutional changes to improve and strengthen P/CEnR and institutional health inequities in the long term.

Through this work, the Champion Team became part of a multi-institutional community of practice with UNM, Stanford, and MSM, which has provided a forum to continuously enhance mutual learning around practices and policies related to P/CEnR. The Champion Teams from all three institutions had the opportunity to meet in person in New Mexico in spring 2023 to share challenges, lessons learned, and ideas for future collaborations. Most recently, partners from MSM provided a keynote address on applying equity principles to practice when conducting CBPR at OCOE’s Pathways to Equity Symposium at Fred Hutch in Seattle, WA [22]. In the long term, this groundwork will inform the Consortium’s plans to address structural and institutional barriers in order to advance P/CEnR and cancer health equity.

The qualitative data (leadership interviews, investigator focus group, and community member/patient advocate focus group) provided a full picture of current practices and perceptions around community and patient engagement in research. Leaders and investigators felt that some work was being done in this area, such as some initiatives and resources for DEI and community and patient engagement. They acknowledged there was room for improvement and recognized the importance of building trust and relationships to address the long-term health and well-being of community, as well as ongoing monitoring by the Consortium for accountability and identification of resources needed for this work.

Community members and patient advocates felt the Consortium still has a long way to go to engage community and patients proactively and equitably in research. They discussed power dynamics between providers/researchers and patients and highlighted the importance of research teams incorporating community and patient voices throughout the research process so that P/CEnR can improve their research outcomes. They brought up the need for P/CEnR training for providers/researchers, community members, and patient advocates, as well as compensation for patient advocate time and expertise. Sharing post-trial data with the communities involved in the research was also identified as an unfulfilled need.

The timing of the Champion Team’s participation in E2PLUS overlapped with several key initiatives with the Consortium, and an initiative at the state level, which provided opportunities to influence health equity-oriented research and care and advance the Champion Team’s targets of change for action to strengthen equity-based P/CEnR. First, in 2020, Fred Hutch created an Office of DEI and appointed a Vice-President and Chief DEI Officer. Second, ongoing dialog within the UW led to the launch of the Center for Anti-Racism and Community Health within the UW SPH in the fall of 2021. This provided the opportunity to codevelop a structure to provide opportunities for consultation, collaboration, partnership, advocacy, activism, and shared decision-making with Black and Indigenous communities as a form of reparations for legacies of slavery, genocide, and assimilation. Third, in January 2022, the Consortium launched a 6-month Task Force to examine inclusion and equity in research to identify patient-centric barriers to participation in clinical trials. Fourth, in the spring of 2022, adult-focused cancer care was focused within the Consortium by bringing together FHCRC, SCCA, and UW Medicine’s cancer program into a single, independent nonprofit organization called Fred Hutch. As a result, cancer research collaborations were also further cemented between Fred Hutch and UW due to this close relationship. Finally, in February 2023, House Bill 1745 was introduced into the WA State Legislature, signed in May, and passed in July. HB 1745 aims to improve diversity in clinical trials by: 1) improving data quality for diverse demographic groups in clinical trials; 2) identifying barriers faced by underrepresented communities and encourage their participation; 3) enhancing transparency of demographic data; and 4) requiring offering culturally specific recruitment materials and trial information in languages other than English.

During the last E2PLUS workshop, the Champion Team identified four strategies to address urgent and persistent institutional health inequities: 1) increase participation of UR groups at all levels of institutional leadership and institutional advisory boards; 2) create an Office of Patient Engagement, with a major focus on UR patients, to train and support patients who participate in institutional initiatives and advise research teams as patient advocates; 3) expand community engagement training, resources, and institutional commitment, including nonresearch engagement focused on community-identified social and health needs; and 4) establish an umbrella entity for health equity efforts across the Consortium.

The Consortium’s involvement in E2PLUS has provided time for introspection, identification of strategies, and a call to action. To work on “increased participation of UR groups at all levels of institutional leadership and institutional advisory boards,” the Fred Hutch Office of Faculty Affairs and Diversity, founded in 2022 to increase the numbers of and support the success of UR faculty and trainees, is tracking metrics around the participation of UR groups in the workforce and institutional advisory boards. UR patients have joined the Consortium’s External Advisory Board and UR community leaders have joined the Fred Hutch Board of Directors and Board of Advisors, ensuring patient voices are integrated into leadership and governance across Fred Hutch. Additionally, Champion Team members played a significant role in helping a new institutional precision oncology initiative establish a diverse patient advisory board. Regarding the “creation of an Office of Patient Engagement to train and support patients who participate in institutional initiatives and advise research teams as patient advocates,” institutional support was made available to recruit a manager to lead this work, who will start in July 2024. This program will be part of Fred Hutch’s Office of Patient Experience.

To support the expansion of “community engagement training, resources and institutional commitment,” the Consortium’s Community Action Boards across WA participated in Consortium strategic planning. The OCOE now coordinates the annual Community Grants Program with the Community Benefit program by pooling financial resources to more equitably fund community-identified social and health needs. In addition, OCOE and several other Fred Hutch departments collaborated with a patient advocate and Champion Team member on the development of a series of videos that highlight Black/African-American patients and Consortium providers to spark conversations around racism and unconscious bias in healthcare [23]. Further, Fred Hutch has received funding from the Andy Hill Cancer Research Endowment Fund, a fund created under state law to appropriate a state match of up to $10 million annually to fund cancer research in WA [24], to pilot “GUIDE: Guiding participation toward Understanding, Inclusion, Diversity and Equity for cancer clinical trials,” a program to address health-related social needs and the financial burden of participation in clinical trials. Finally, in order to have an umbrella entity for health equity efforts across the Consortium, the Health Equity Steering Committee (HESC) was formed in 2023 and institutional support was provided to recruit a project coordinator for tracking and reporting of this work.

As part of the ongoing work of Fred Hutch to become an antiracist institution, in January 2022, the Fred Hutch Director convened a 6-month, Task Force on Inclusion and Equity in Research (Task Force) to advise Consortium and institutional leadership about opportunities for better patient care and more robust, inclusive, and equitable approaches to research studies and clinical trials. Seven Champion Team members were key members of this Task Force or presented crucial data to the Task Force.

The Task Force aligned with facilitators and barriers identified through the E2PLUS project and became the vehicle to mobilize E2PLUS strategies within the Consortium. After the Task Force shared recommendations (Table 2) with Consortium leadership in late summer of 2022, the Task Force sunset as planned, and the Consortium-wide HESC was appointed in 2023 to oversee the planning and implementation of the recommendations.

Three Champion Team members joined the HESC. The HESC is developing a 3-year health equity strategic plan to foster the organizational alignment needed to address health inequities under three pillars in 1) research, 2) patient experience and outcomes, and 3) community engagement and outreach. The HESC will be responsible for the direction, prioritization, and approval of health equity initiatives and measurements of success. They will make strategic adjustments when goals are missed, as well as prioritize health equity-related resource requests.

The Consortium aspires to advance cancer research that is meaningful and accessible to all. Current work is shifting policies and practices thereby strengthening institutional infrastructure to enable inclusion and equity in community partnerships, patient care, meeting patient care needs, access to clinical trials, and ongoing monitoring for accountability and identification of resources needed for this work. As a result of the Consortium’s involvement in E2PLUS, over the long term, we expect to see an enhanced capacity of Consortium faculty and staff to engage patients and community members in P/CEnR to address cancer health inequities.

### Limitations

There are some limitations to this work. The sample size for qualitative data was small. Participants in the E2PLUS project were “willing and ready” to engage in this work. Future activities should include a broader range of individuals who can contribute to further strengthening the capacity for P/CEnR. While participating in E2PLUS has helped identify actions to address cancer health equity, there is a need to identify metrics to show if strategies to increase P/CEnR across the Consortium are working.

## Conclusion

Participating in E2PLUS was the catalyst for institutional change that helped the Consortium identify and begin to address structural issues impeding equity in cancer care and outcomes in WA. Having a Champion Team that included institutional representatives from the Consortium and members from the community, including cancer survivors and patient advocates, was critical to this work. While pockets of health equity efforts were already happening across Consortium institutions, E2PLUS brought many leaders and staff together to plan how to work together in meaningful and synergistic ways. In addition, engaging programs from across the Consortium as well as community partners helped establish understanding and in turn, buy-in about the need for infrastructure change.

An overarching theme identified through this work was the need to create room and resources for communities/patients to meaningfully engage with the Consortium in the pursuit of cancer health equity. Between E2PLUS and the Task Force documenting needs/gaps and ongoing initiatives to meet those needs/gaps, this new awareness of cross-cutting efforts allowed leadership to put resources strategically into multiple efforts at once and with plans to enable more in the coming years. The HESC will facilitate and document ongoing and future initiatives and will report progress to leadership on a regular basis. The Consortium’s participation in E2PLUS provided vital technical assistance from UNM for this work, cross-institutional learning with Stanford and MSM through the multi-institutional community of practice, and helped OCOE drive the development of capacity for P/CEnR, with an emphasis on UR Washingtonians.
